# The Benefit of Combining Neuronal Feedback and Feed-Forward Control for Robustness in Step Down Perturbations of Simulated Human Walking Depends on the Muscle Function

**DOI:** 10.3389/fncom.2018.00080

**Published:** 2018-10-09

**Authors:** Daniel F. B. Haeufle, Birgit Schmortte, Hartmut Geyer, Roy Müller, Syn Schmitt

**Affiliations:** ^1^Multi-Level Modeling in Motor Control and Rehabilitation Robotics, Hertie Institute for Clinical Brain Research and Center for Integrative Neuroscience, Eberhard-Karls Universität Tübingen, Tübingen, Germany; ^2^Biomechanics and Biorobotics, Stuttgart Center for Simulation Sciences (SC SimTech), University of Stuttgart, Stuttgart, Germany; ^3^Robotics Institute, Carnegie Mellon University, Pittsburgh, PA, United States; ^4^Institute of Sport Science, Friedrich Schiller University of Jena, Jena, Germany; ^5^Department of Neurology and Department of Orthopedic Surgery, Klinikum Bayreuth GmbH, Bayreuth, Germany

**Keywords:** musculo-skeletal model, Hill-type muscle, simulation, forward-dynamics, perturbation, reflex

## Abstract

It is often assumed that the spinal control of human locomotion combines feed-forward central pattern generation with sensory feedback via muscle reflexes. However, the actual contribution of each component to the generation and stabilization of gait is not well understood, as direct experimental evidence for either is difficult to obtain. We here investigate the relative contribution of the two components to gait stability in a simulation model of human walking. Specifically, we hypothesize that a simple linear combination of feedback and feed-forward control at the level of the spinal cord improves the reaction to unexpected step down perturbations. In previous work, we found preliminary evidence supporting this hypothesis when studying a very reduced model of rebounding behaviors. In the present work, we investigate if the evidence extends to a more realistic model of human walking. We revisit a model that has previously been published and relies on spinal feedback control to generate walking. We extend the control of this model with a feed-forward muscle activation pattern. The feed-forward pattern is recorded from the unperturbed feedback control output. We find that the improvement in the robustness of the walking model with respect to step down perturbations depends on the ratio between the two strategies and on the muscle to which they are applied. The results suggest that combining feed-forward and feedback control is not guaranteed to improve locomotion, as the beneficial effects are dependent on the muscle and its function during walking.

## 1. Introduction

One way of trying to better understand human motor control is to simulate human walking with neuro-musculoskeletal models. These models generate locomotion by the interaction of their neuronal control and musculoskeletal dynamics, allowing to explore the consequences of different control approaches. Several such control approaches have evolved over time. One approach to modeling the control of human walking is to generate all muscle stimulation signals based on reflexes (Geyer and Herr, 2010; Song and Geyer, 2015), which relate proprioceptive feedback of muscle sensory organs to alpha-motor neurones (αMN) in the spinal cord. Another approach is to rely on central pattern generators (CPGs) as the primary source of muscle stimulation (Taga et al., 1991; Hase et al., 2003). CPGs are low-level circuits that can generate rhythmic patterns without external feedback stimuli, representing feed-forward control. Most models combine feed-forward and feedback control to produce walking, either by combining CPGs with general muscle reflexes (Ogihara and Yamazaki, 2001; Hase et al., 2003; Paul et al., 2005; Aoi et al., 2010; Kim et al., 2011; Dzeladini et al., 2014; van der Noot et al., 2015), or by using feed-forward signals (potentially forming a CPG) to set references for specific mono-synaptic stretch reflexes (Günther and Ruder, 2003). All of these models have in common, that no higher level multi-synaptic networks are required for steady-state walking and that they are robust to some perturbations in the environment.

The combination of feed-forward and feedback control is to some extend inspired by experimental observations and physiological evidence. Experiments demonstrate the contribution of reflexes as direct feedback to the muscle stimulation in animal (e.g., Donelan and Pearson, 2004) and human locomotion (Schneider et al., 2000; Grey et al., 2007; van der Linden et al., 2007). On the other hand, the existence of central pattern generators in the spinal cord providing rhythmic feed-forward control signals has been shown in animal (Orlovsky et al., 1999; Ijspeert, 2008) and discussed for human locomotion (MacKay-Lyons, 2002; Minassian et al., 2017). Experimental evidence further suggests that both, feedback and feed-forward commands simultaneously contribute to muscle stimulation in human locomotion (McDonagh and Duncan, 2002; Müller et al., 2015).

The co-existence of both signal types raises the question of what the benefit of their combination is. Kuo (2002) showed in a computer simulation with a simple model, consisting of a pair of antagonistic muscles driving one segment, that the combination of feed-forward and feedback signals allows to increase stability of rhythmic movements. This observation was confirmed in a very simple muscle-driven hopping simulation (Haeufle et al., 2012), where a linear combination of both signals allowed to reject a perturbation in ground level within one hopping cycle. The major reason for the benefit was, that a certain feed-forward contribution allowed to increase the muscle stimulation in preparation for ground contact. If the ground contact was delayed due to a drop in ground level, the leg muscle was already more active, and hence, could generate larger breaking forces early in the stance phase. This allows to partially compensate for the neuronal delays and offers a feed-forward strategy to prepare for ground contact. Although these studies show that the combination of feed-forward and feedback increases the stability and robustness of dynamic repetitive movements, it remains unclear whether these findings extend to more complex tasks such as human walking.

Here we investigate if the combination of feed-forward and feedback signals improves the robustness of walking, a more complex but highly relevant human movement. To this end, we resort to a previously published model of human walking (Geyer and Herr, 2010), which in its original form solely relies on spinal feedback control to generate walking. We extend this feedback control with feed-forward muscle stimulation patterns and study the change in the model's reaction to step down perturbations as a function of the ratio between the two control strategies. This highlights especially the role of feed-forward for unexpected touch-down conditions. We find that the general notion of an advantage of the combination of feed-forward and feedback control does not hold for all muscles, as they serve different purposes in walking.

## 2. Methods

### 2.1. Combining feedback and feed-forward

**Figure 1:**
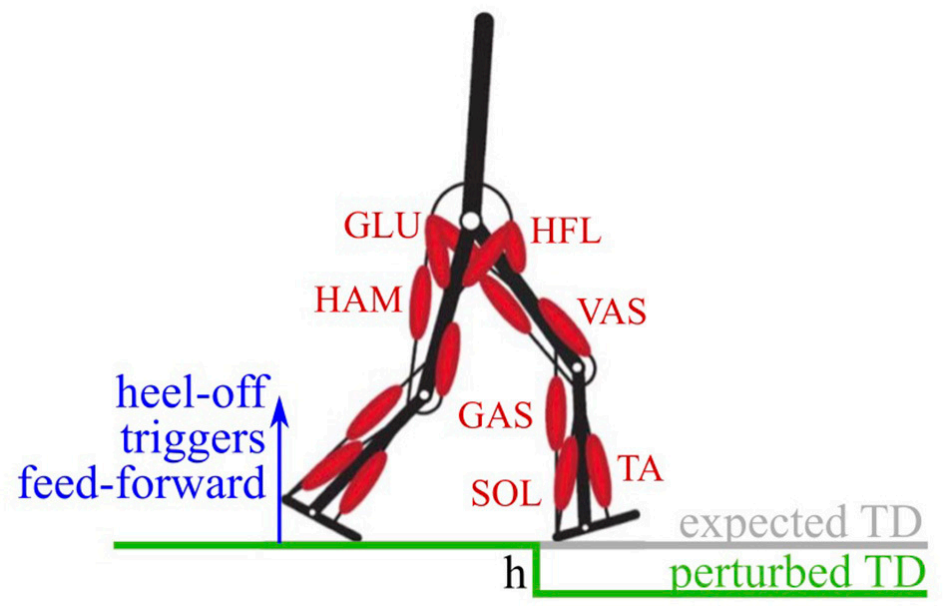
The walking model has two legs, actuated by seven Hill-type muscle-tendon units per leg (Geyer and Herr, 2010). Feed-forward signals in the model are triggered on the heel-off of the contralateral leg. A perturbation in the ground level of drop-height *h* causes a delayed touch down (perturbed TD) with respect to the expected touch down.

We investigated the combination of feedback and feed-forward signals in the walking model of Geyer and Herr (2010). The model predicts the saggital kinematics and dynamics of human walking, as well as the muscle activities. It has two legs, each composed of three rigid segments (foot, shank, thigh), and an upper body connected by six joints idealized as single degree of freedom hinge-joints (Figure 1). The joints are actuated by 14 Hill-type muscle-tendon units considering the contraction dynamics of active muscle fibers, parallel passive muscle tissue, tendon elasticity, and first-order activation dynamics. For this study, we only modified the neuronal control, all other bio-mechanical properties were kept as in the original publication (see Geyer and Herr, 2010 for more details).

**Figure 2:**
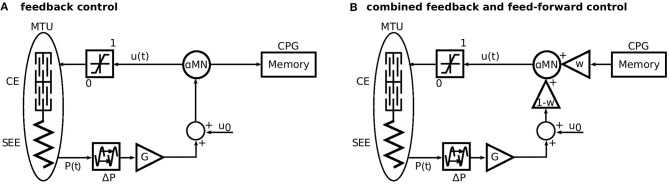
Sketch of the approach to generate the feed-forward control signal and the combination of both control schemes. **(A)** First, the model is driven only by a reflex-based feedback control. Here, the stimulation to the alpha motor neurone (αMN) is recorded to a memory. **(B)** For the simulations with combined control strategies, the recorded stimulation signal is played back as a feed-forward signal. The feed-forward signal is triggered, when the heel of the contralateral leg leaves the ground (Figure 1). This represents the last known state of the environment. The parameter *w* linearly scales between feedback (*w* = 0) and feed-forward (*w* = 1) control. Modified from Geyer et al. (2003).

In the original formulation of this model, the muscle stimulation was generated solely by proprioceptive feedback. That is, the feedback stimulation signals $u_{m}^{FB}\left(t\right)$ for each muscle *m* were based on proprioceptive sensory signals *P*(*t*) e.g., from muscle spindles, Golgi tendon organs, or the vestibular organ. These signals are subject to neuronal delay Δ*P* and multiplied by synaptic gains *G* (Figure 2A). The proprioceptive feedback control strategy was described in detail by Geyer and Herr (2010).

We generated feed-forward signals $u_{m}^{FF}\left(t\right)$ from the existing control, by recording the time trace of the original feedback-generated muscle stimulation $u_{m}^{FB}\left(t\right)$ in unperturbed steady-state walking (Animation 9, see Electronic Supplementary Material). This way, a feed-forward muscle stimulation signal can be derived without the need for optimization or models of a central pattern generator (in contrast to Haeufle et al., 2012 or Dzeladini et al., 2014, respectively). The feed-forward signals were triggered by the heel take-off of the contralateral leg, where the leg of interest is at the end of its swing phase. This represents the last point where the biological systems can estimate the ground level without the aid of visual feedback and cause the feed-forward pattern to stimulate the muscles as if the ground was level and no perturbation is to be expected.

To investigate the combination of feedback and feed-forward, we linearly combined the two signals as an input to the α-motor neuron (Figure 2B):

(1) $$u_{m}(t)=(1-w_{m})\cdot u_{m}^{FB}(t)+w_{m}\cdot u_{m}^{FF}(t).$$

This leaves us with one free parameter for each muscle: the weighting factor *w*_*m*_ which quantifies the relative contribution of the feed-forward signal, with *w*_*m*_∈[0, 1].

### 2.2. Effect of the combination in a simple hopping example

The effect of the combination can be best demonstrated with a simple example. For this, we exemplarily combine a previously published hopping model (Geyer et al., 2003) consisting only of one knee extensor muscle with the proposed linear combination of feed-forward and feedback (Haeufle et al., 2012). As this hopping model has only one muscle and investigates only the rebounding behavior, the effect can be demonstrated more clearly.

**Figure 3:**
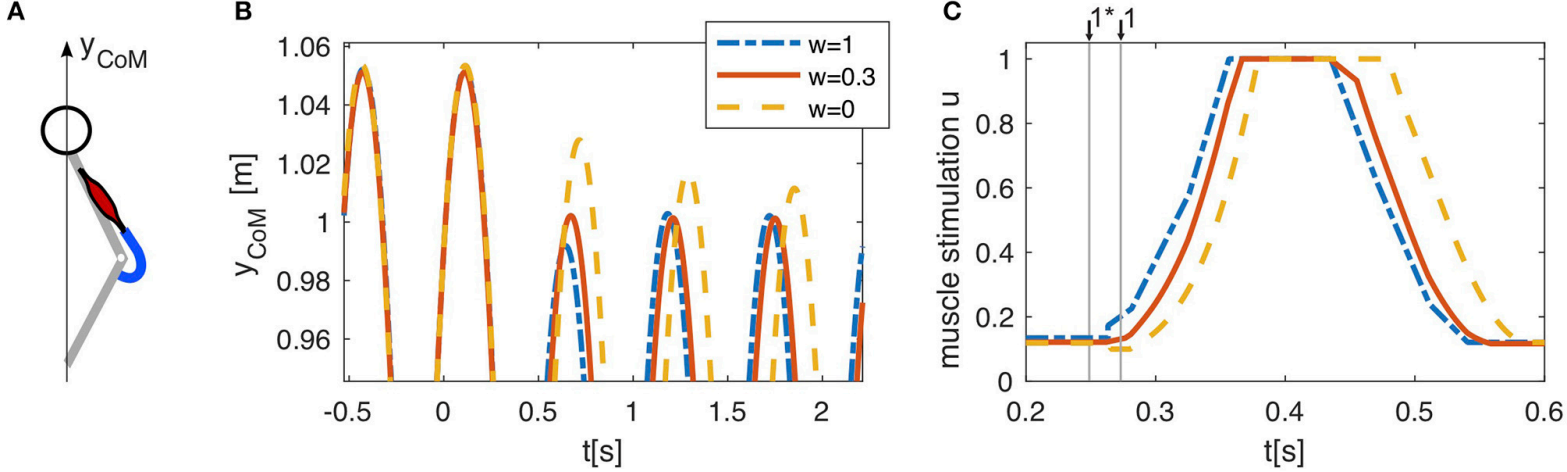
**(A)** In a reduced hopping model (Geyer et al., 2003), only one muscle (knee extensor m. vastus) generates the anti-gravity forces. **(B)** Pure feed-forward (*w* = 1) and pure feedback (*w* = 0) control both lead to stable hopping. After a drop in ground level height (*h* = −0.05m, at *t* = 0s), the center of mass (CoM) reaches a periodic hopping height within two hopping cycles for the feed-forward control and within several cycles for the feedback control. Combining both control strategies (*w* = 0.3) allows to compensate for the perturbation within one hopping cycle. Shown here is only the upper part of the CoM trajectory to expand details of the adaptation to the perturbation. **(C)** The feedback muscle stimulation (*w* = 0) is generated by positive force feedback which causes rapidly rising stimulation after touchdown with a neuronal delay of ∆*P* = 0.015s. In presence of a perturbation, the feedback based stimulation (*w* = 0) can only increase after the actual touchdown, when the muscle is stretched and generates forces in the interaction with the ground. A feed-forward pattern triggered to the last touchdown (*w* = 1) starts stimulating the muscle shortly after the expected touchdown for level hopping ↓1* and is therefore already more active at the actual touchdown ↓1 in the case of a drop down perturbation (*h* = −0.05m, at *t* = 0s). Linearly combining both strategies (*w* = 0.3) results in an intermediate behavior, which is beneficial to the stability of the hopping pattern.

The hopping model of Geyer et al. (2003) was driven only by one muscle-tendon unit (Figure 3A). In unperturbed periodic hopping, positive force feedback generates a feedback signal *u*^*FB*^(*t*) which is low in the beginning of the stance phase and rises with increasing muscle force during the stance phase (see Figure 3C). This signal was recorded and used as a feed-forward command *u*^*FF*^(*t*) (Figure 2). The beginning of the feed-forward signal was triggered on the take-off, the last known state of the environment. Combining both signals according to Equation (1) (Figure 2B) has no effect on unperturbed hopping, as both signals *u*^*FB*^(*t*) and *u*^*FF*^(*t*) generate the same muscle stimulation. However, if we introduce a perturbation by lowering the ground level, the model behavior changes. The drop in ground level causes a delayed ground contact with higher kinetic energy of the center of mass leading to higher forces in the muscle. For pure feedback, the neuronal response to the perturbation only happens after the delayed ground contact resulting in a delayed sensor signal *P*(*t*) and hence, a delayed αMN stimulation (Figure 3C). For pure feed-forward, neither timing nor amplitude change: the feed-forward pattern does not consider the increased duration of the flight phase as it is triggered on the last unperturbed take-off and it does not consider the increased muscle strain. Hence, the muscle is already in a more active state at the instance of the delayed ground contact and does not rise as high (Figure 3C). Combining both according to Equation (1) allows to combine both effects. This can be exploited to increase the stability and reject the perturbation within one hopping cycle (Figure 3B).

This application of the control method to the simple hopping model demonstrates the potential benefit, as it would generally be expected. In the following, we will investigate how this concept generalizes to walking. Walking is more complex and the model has to achieve more movement requirements than just stabilizing vertical rebounding behavior, e.g., stabilizing the trunk, generating ground clearance in swing phase, generating push-off for swing leg acceleration, etc.

### 2.3. Model analysis

To investigate the robustness of the walking model, we implemented ground perturbations by modifying the ground level

(2) $$y_{\text{0}}\text{= 0m + }h\text{,}$$

where *h* is the perturbation height. For the majority of the simulation experiments, the perturbation height was negative, meaning a drop in ground level. We define a walking pattern to be robust, if the model successfully continues to walk for at least 10 s (approximately 20 steps) after the perturbation. In general, this means that the model returned to steady state walking after the perturbation. Although the focus of this study was on step down perturbations, we also investigated small step up perturbations up to the limit of ground clearance (*h* = 0.014m), as well as walking on downhill slopes.

For the analysis of the model, we changed only the weighting factors *w*_*m*_ between feed-forward and feedback and investigated the robustness by increasing the step down perturbation height *h*∈{−0.01, −0.02, …} m. First, we modified the feed-forward contribution only for single muscles (*w*_*m, i*_∈{0, 0.1, 0.2, …, 0.9, 1}) while all other muscles were driven by pure feed-forward (*w*_*m*, exept i_ = 0). In a second step, we looked into simultaneous feed-forward contributions to several muscles. For this, we performed a systematic search, where we investigated all linear combinations of two muscles with varying feed-forward contributions, while the other five muscles per leg were still only driven by pure feedback signals.

## 3. Results

The reference model with pure feedback control (*w*_*m*_ = 0 for all muscles) tolerates height perturbations of up to *h* = −0.03m. Larger perturbations lead to a collapse in the first step after the ground height change (Anim. 0).

### 3.1. Control combination applied to individual muscles

**Figure 4:**
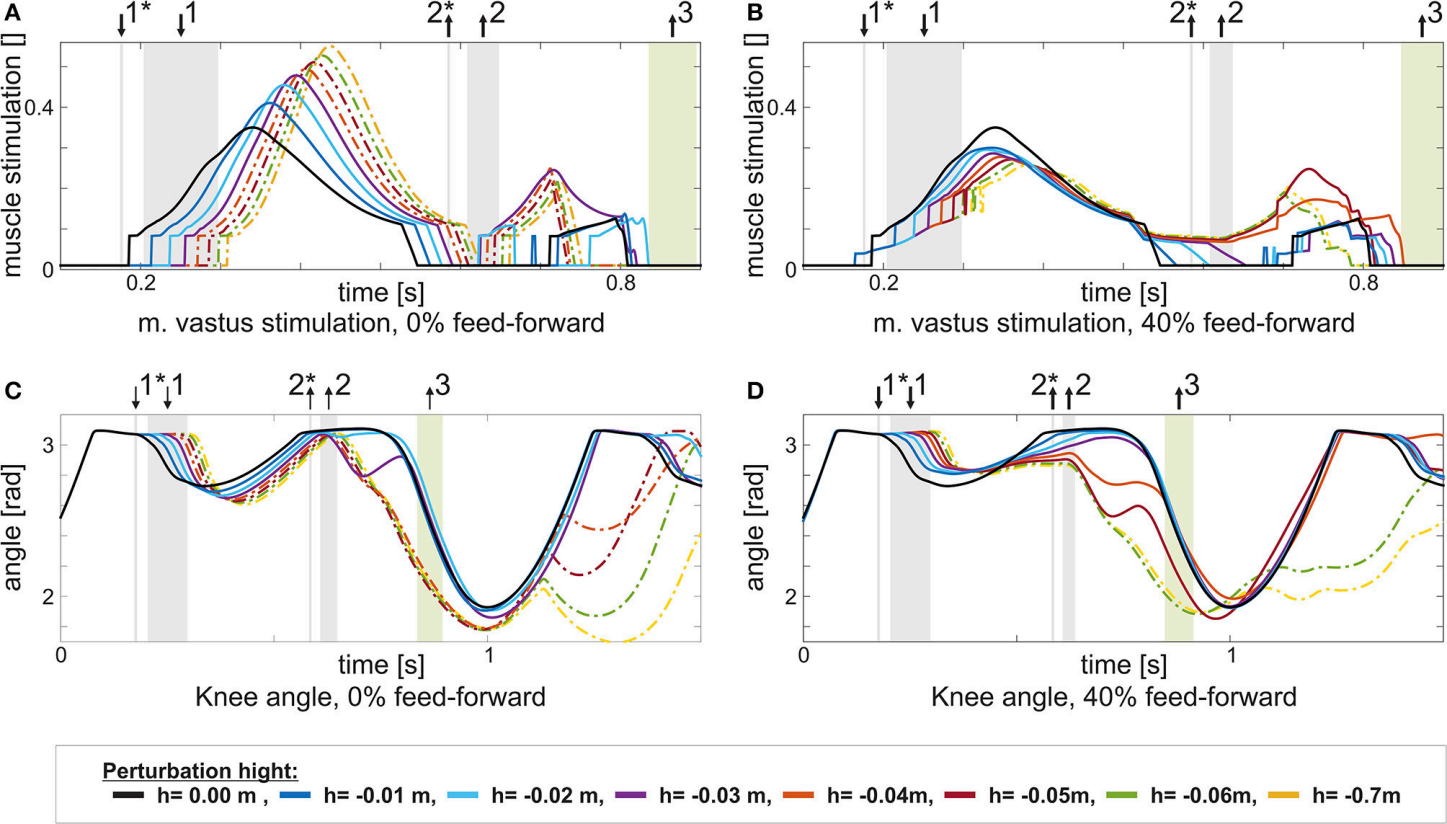
This figure shows the change in stimulation *u*_VAS_ and knee joint angle for increasing perturbation height *h* with the same feed-forward / feedback combination: 0% in the left column, and 40% in the right column. Step size increases from *h* = 0m (black) to *h* = −0.07m. Heel contact is indicated by ↓1, heel-off by ↑2, and toe-off by ↑3. Heel-contact and heel-off marked with *represent the reference case (0%, level walking). **(A)** It becomes visible, how an increased perturbation first delays and in consequence increases m. vastus stimulation, if no feed-forward contribution is present. **(C)** The delayed stimulation causes the knee to further flex in the stance phase. In consequence, the model stumbles. **(B)** For the combined control (40%), the delay is partially compensated by the triggered feed-forward stimulation pattern. **(D)** Hence, the knee is less flexed and stumbling is prevented for larger perturbations.

Adding a feed-forward contribution to either the knee or the ankle extensor muscles increases walking robustness. For instance, with a feed-forward contribution of *w*_VAS_ = 0.4 to the m. vastus (VAS), the model can tolerate larger step down perturbations of up to *h* = −0.05m. The effect of the feed-forward contribution is similar to what was shown in section 2.1 for the extensor muscle in the hopping model: A pure feedback contribution can act only after ground contact, delaying the onset of muscle stimulation in the case of a step down perturbation (Figure 4A). Adding the feed-forward contribution generates an earlier onset of the muscle stimulation, reducing muscle strain and increasing robustness (Figure 4B, between ↓1* and ↓1). Similarly, a feed-forward contribution to either the m. soleus (SOL, *w*_SOL_ = 0.2) or the biarticular m. gastrocnemius (GAS, *w*_GAS_ = 0.6) increases the model tolerance to larger step down perturbations (up to *h* = −0.07m in case of GAS, more than twice the maximum perturbation of the feedback controlled reference model). However, for the other muscles, no level of individual feed-forward contribution increases walking robustness.

Furthermore, for all muscles the level of feed-forward contribution affects walking robustness. For example, the robustness of VAS increases with increasing feed-forward contribution until *w*_VAS_ = 0.4 (Table 1, Anim. 1). Larger feed-forward contributions quickly diminish robustness, and no stable walking is possible for *w*_VAS_≥0.6 (Anim. 2). The muscle group most sensitive to the level of feed-forward contribution are the hip flexors HFL, for which *w*_HFL_≥0.3 already destabilizes the walking pattern. On the other extreme, GAS requires relatively high contributions *w*_GAS_≥0.5 (Anim. 4) and is the only muscle that can be stimulated by a pure feed-forward signal (*w*_GAS_ = 1) without reducing walking robustness. In effect, the level of feed-forward contribution has to be chosen carefully for each muscle and, in most muscles, cannot be too large without destabilizing the walking pattern, suggesting that, within the spinal control structure proposed by the walking model, a CPG-like drive as the sole provider of muscle stimulations fails to generate walking.

**Table 1:** The table shows successful (✓) and unsuccessful (×) walking simulations for variations in perturbation height *h* and feed-forward contribution ω in m.vastus (VAS).

		**Feed-forward** ω **[%]**						
	**VAS**	**0**	**10**	**20**	**30**	**40**	**50**	**60…100**
h [m]	0.00	✓	✓	✓	✓	✓	✓	×
	−0.01	✓	✓	✓	✓	✓	✓	×
	−0.02	✓	✓	✓	✓	✓	✓	×
	−0.03	✓	✓	✓	✓	✓	✓	×
	−0.04	×	×	×	✓	✓	×	×
	−0.05	×	×	×	×	✓	×	×
	−0.06…−0.15	×	×	×	×	×	×	×

*See also Animation 1 is the case ω = 40, h = −0.05; Animation 2 is the case ω = 60, 100, h = 0.00*.

### 3.2. Control combination applied to muscle groups

A systematic search with feed-forward signals applied to several muscles reveals that some combinations can further improve walking robustness while others destabilize walking or change the entire walking pattern. For instance, combining the most robust case for VAS (*w*_VAS_ = 0.4) with simultaneous feed-forward contributions to the hip muscles shows that it improves robustness with GLU (Table 2b, Anim. 5) but not with HFL (Table 2a), which destabilized walking for *w*_HFL_≥0.3. On the other hand, a combination of SOL (*w*_SOL_ = 0.2) and HFL (*w*_HFL_ = 0.4) is robust to perturbations of up to three times the pure feed-forward robustness (*h* = −0.09m, Anim. 7). Here, the recovery after the perturbation takes several steps, but the steady walking pattern still appears natural. In contrast, the most robust case was found for a combination with GAS (*w*_GAS_ = 0.8) and GLU (*w*_GLU_ = 0.1), which increased the step tolerance to *h* = −0.12m but resulted in a slower and unnaturally looking steady walking pattern (Anim. 8).

**Table 2:** The tables **(a,b)** show successful (✓) and unsuccessful (×) walking simulations if two muscles have a feed-forward contribution.

		ω **[%]**					ω **[%]**			
	**VAS**	**40**	**40**	**40**		**VAS**	**40**	**40**	**40**	**40**
	**HFL**	**10**	**20**	**30**		**GLU**	**10**	**20**	**30**	**40**
h [m]	0.00	✓	✓	×	h [m]	0.00	✓	✓	✓	✓
	−0.01	✓	✓	×		−0.01	✓	✓	✓	✓
	−0.02	✓	✓	×		−0.02	✓	✓	✓	✓
	−0.03	✓	×	×		−0.03	✓	✓	✓	✓
	−0.04	✓	✓	×		−0.04	×	✓	✓	✓
	−0.05	✓	✓	×		−0.05	✓	✓	✓	×
	−0.06	×	×	×		−0.06	×	✓	✓	✓
	−0.07	×	×	×		−0.07	×	×	×	×
(a) VAS and HFL combination					(b) VAS and GLU combination					

*See also Animation 5 is the case VAS 40 GLU 20, h = −0.06*.

A more in depth description of the resulting walking and stimulation patterns can be found in the Electronic Supplementary Material, along with the animations and the model output data.

### 3.3. Other perturbations

The possibility to investigate perturbations with positive heights in this model is very limited. The model has a relatively low ground clearance of only about 0.014 m (see for example Anim. 9). This means, that the maximum perturbation height for positive heights is limited. All such perturbations can directly be compensated by the pure feedback control scheme (w=0, Anim. 10, *h* = 0.012[*m*]). Larger step up perturbations lead to an early ball contact in mid-stance resulting in stumbling, which cannot be compensated by just adding feed-forward signals.

Feed-forward contributions to some muscles also allows to walk down steeper slopes than for pure feedback control, where the maximum slope angle is $\alpha_{\max}=3.4^{{}^{\circ}}$. The maximum slope angle $\alpha_{\max}=5.7^{{}^{\circ}}$ for any contribution of m. vastus is achieved with *w*_VAS_ = 0.4 (Anim. 11), which is the same feed-forward contribution which leads to the maximum step down perturbation. Also feed-forward contributions to m. soleus lead to larger maximum slope angles ($\alpha_{\max}=5.7^{{}^{\circ}}$ for *w*_SOL_ = 0.1) while they destabilize the walking if applied e.g., to the hip flexor muscle group ($\alpha_{\max}=2.3^{{}^{\circ}}$ for *w*_HFL_ = 0.4). Overall, the effect is similar to the step down perturbations.

## 4. Discussion

Presumably, a combination of feed-forward and feedback contributions should improve control performance. While this notion has been confirmed for comparably simple neuro-musculoskeletal models and tasks (Kuo, 2002; Haeufle et al., 2012), we find that it may be misleading for more complex behaviors. We introduced step down perturbations (modification of ground contact height) in a human walking model and studied the effect of combining feed-forward and feedback control of muscles to the robustness of walking. Our results show that combining the two control types can improve walking robustness when applied to some muscles but not to others, suggesting the benefit of a control combination cannot be presumed and requires to consider individual muscle function.

### 4.1. Benefit for robustness depends on muscle function

The anti-gravity muscles (VAS, SOL, GAS) of the walking model benefit from adding feed-forward control. One key function of these muscles is to counteract the gravitational force and reversing the body's vertical motion in stance. Although, overall, legs behave similar to elastic springs in steady running and walking (Blickhan, 1989; McMahon and Cheng, 1990; Geyer et al., 2006; Lipfert, 2010), a drop in ground level results in an increased impact velocity and additional kinetic energy (Müller and Blickhan, 2010, e.g., in running) that has to be dissipated or redirected by the muscles in the leg (Kalveram et al., 2012). As demonstrated for hopping in section 2.1), a feed-forward control for an anti-gravity muscle can ensure a rising stimulation in anticipation of a larger impact due to the drop in ground level. In contrast, a stimulation based purely on feedback control does not sufficiently stimulate the muscle early in the stance phase (Haeufle et al., 2012). In humans as well as in the walking model, the stimulation of VAS (Figure 4) rises in the initial stance phase (Hof et al., 2002; Geyer and Herr, 2010). With a pure feedback control, however, this stimulation can only increase after ground contact (Figure 4A vs. Figure 4B). In the case of an unexpected drop in ground level, the knee bends more than usual (Figure 4C, between ↓^1^ and ↓^2^) due to the additional kinetic energy and the walking pattern cannot be recovered. With the early onset of the feed-forward stimulation, the leg generates larger braking forces dissipating the additional energy and avoiding excessive knee bending (Figure 4D, between ↓^1^ and ↓^2^). As a result, the maximum perturbation height increases (Table 1).

The bi-articular m.gastrocnemius partially works as anti-gravity muscle like m. soleus at the ankle. However, it also flexes the knee. Adding feed-forward to m.gastrocnemius therefore influences knee and ankle joint and has two consequences: Firstly, in the perturbed step (e.g., *h* = −0.07m), the original feedback model flexes the swing leg's knee less compared to level walking and the stance phase is not capable of keeping the CoM high enough above ground. Combined, this results in an early ground contact of the swing leg's foot (too short stride length) and the model stumbles. Adding feed-forward to m. gastrocnemius increases swing leg knee flexion and therefore prevents stumbling. The second consequence is, that the relatively high feed-forward contributions in m. gastrocnemius, which actually improve robustness up to *h* = −0.07m (for *w*_GAS_ = 0.8), also result in a modification of the gait pattern with longer stance phases and early heel lift-offs. This can be attributed to the bi-articular function of m. gastrocnemius which also coordinates the energy release in the push-off phase (Lipfert et al., 2014) where the timing between ankle and knee joint is crucial (van Ingen Schenau et al., 1987). The feed-forward contribution perturbs this coordination.

Other muscles do not benefit from adding feed-forward control. For instance, feedforward contributions to the hip muscles (HFL and GLU) rather tend to destabilize the model. These muscles primarily serve a different function in walking, maintaining trunk balance, which requires a reactive control scheme (Horak and Nashner, 1986; Tang et al., 2012; Müller et al., 2017). A higher feed-forward contribution reduces the ability of these muscles to adequately react to a perturbed orientation of the trunk, especially for the HFL, which in normal locomotion becomes active in late stance. Only in combination with other muscles, a small feed-forward contribution to GLU improved robustness by partially compensating for the increased forward lean of the trunk early in the perturbed stance phase. Experiments even suggest, that a feed-forward strategy may be employed to erect the trunk in case of an unexpected drop (Müller et al., 2014, 5.5deg for a *h* = −0.1 m drop). In combination with m. vastus' feed-forward stimulation compensating for the additional kinetic energy, a slight m. gluteus feed-forward stimulation (*w*_GLU_ = 0.2 *w*_VAS_ = 0.4) to erect the trunk after the perturbation allows to compensate for a maximum drop height of *h* = −0.06 m.

Similarly, muscles which are active in the swing phase (hip flexor group, hamstrings, and tibialis anterior) do not benefit from a feed-forward contribution in the model. Similar to the trunk stabilization in stance, the leg in swing requires reactive control to achieve proper foot placement, which is critical for maintaining gait stability (Pratt et al., 2006; Hof, 2008). A feedforward contribution triggered at heel-off of the contralateral leg is likely out of sync once the ipsilateral leg enters the swing phase after perturbations. Resetting the feedforward contribution at characteristic transitions in the gait may resolve this issue and has been suggested in the literature for CPGs (Righetti and Ijspeert, 2006; Aoi et al., 2010; Aoi and Tsuchiya, 2011; Li et al., 2017).

### 4.2. Robustness may be improved by more complex models for the signal combination

We expect that refinements of the feed-forward contributions can improve walking robustness. The feed-forward patterns we tested, were recorded signals as generated by the original feedback scheme. We expect that in human control such feed-forward patterns are learned and optimized by the organism based on the interaction with the feedback control. In addition, the simple linear combination of the control contributions is likely oversimplifying the actual situation. For instance, it is known that the signal contributions are modulated during the walking cycle, as indicated by the H-reflex amplitude modulation of the ankle extensors (e.g., Crenna and Frigo, 1987; Schneider et al., 2000). Such a modulation over the gait cycle of the contributions of different signal types may increase the benefit of a combined feed-forward and feedback control (Song and Geyer, 2015). For instance, GLU could in this way prepare the trunk orientation by feed-forward control around impact and correct the orientation during the late stance phase by pure feedback. All these extensions may allow to bring the result closer to the commonly expected benefit of feed-forward control to robustness.

### 4.3. Generalization of the concept to other perturbations

The possibility to investigate perturbations with positive heights in this model is very limited, as the normal walking pattern has only about 0.014m ground clearance (see for example Anim. 9). They can directly be compensated by the pure feedback control scheme (w = 0, Anim. 10, *h* = 0.012[*m*]).

The results for drop down perturbations generalize to walking down a slope. Here, also, the feed-forward contribution to the anti-gravity muscles is beneficial and allow to compensate for the additional kinetic energy in each step. However, the improvement we found was not as consistent as for the step down perturbations, i.e., some flatter slopes may not be compensated for. This means that an improvement may be achieved, however it may require online tuning of the weighting and may not be possible by simply adding the same feed-forward at all times and cases. For the step down perturbations this is unrealistic, as they appear as a sudden unexpected perturbation where no online tuning is possible. Here they have to (and do) work consistently for all perturbations until a certain maximum height. For a slope, such an adaptation may, however, be feasible as the slope may be detected visually or adapted to within the first or second step.

As demonstrated by Song and Geyer (2015), a more recent version of the model allows to simulate other gaits by varying the control parameters of the feedback terms. This may allow to also study different walking patterns and perturbations in the future. Although the found benefit generalizes to slope down walking, other perturbations, like pushes or slips may not necessarily benefit from the combination.

Validating the response to such perturbations experimentally in humans is still difficult. While the general model approach can reproduce a set of perturbation responses quite well (Song and Geyer, 2017), it is difficult to distinguish between feed-forward and feedback contributions in muscle activity recordings (Müller et al., 2015). Only invasive techniques may be able to shed some light on this (Grey et al., 2007). However, we see potential in studying this in exoskeleton supported or prosthetic walking, by varying the contributions in an augmented control scheme.

### 4.4. A purpose of feed-forward and feedback combination beyond robustness?

While it is difficult to distinguish in human walking experiments between feed-forward and feedback contributions, perturbation studies suggest the coexistence of both (Schneider et al., 2000; McDonagh and Duncan, 2002; Grey et al., 2007; van der Linden et al., 2007; Müller et al., 2015). The reported benefit of this coexistence to robustness with respect to external perturbations (Kuo, 2002; Haeufle et al., 2012), however, may not be the only purpose of such control combinations. Alternative suggestions include that internal changes of the system such as changes in muscle force may be better compensated by a combined control scheme (Yakovenko et al., 2004). In addition, Dzeladini et al. (2014) demonstrated an increase in the agility of locomotion. By modulating a feed-forward CPG pattern that was combined with the reflex control scheme of the walking model investigated here, they demonstrated changes in walking speed without the need to re-adjust the synaptic gains of the reflex loops. Other potential benefits include the compensation of inevitable feedback delays by internal feed-forward models (Wolpert and Miall, 1996; Wolpert and Flanagan, 2001) and the reduction of control complexity and effort by relying on feed-forward patterns of low neuronal complexity (Haeufle et al., 2014).

## 5. Conclusion

We have shown with a simulation model of human walking that a simple linear combination of (central pattern generated) feed-forward control and (reflex generated) feedback control can improve walking robustness when compared to isolated feed-forward or feedback control. But we have also shown that this outcome cannot be presumed in general, as the beneficial effects of combining the two strategies are dependent on the muscle and its function during gait. Furthermore, the positive effect was shown for a step down perturbation an may generalize to similar experiments like slope down walking, but not necessarily to other perturbation experiments.

## Author contributions

DH, SSch, and RM conceived of the study and designed the simulation experiments. HG implemented the walking model. BS modified the model and ran the simulations. BS, DH, and RM analyzed the data. All authors contributed to the manuscript. All authors gave final approval for publication.

### Conflict of interest statement

The authors declare that the research was conducted in the absence of any commercial or financial relationships that could be construed as a potential conflict of interest.
