# Supplementary material for: The Benefit of Combining Neuronal Feedback and Feed-Forward Control for Robustness in Step Down Perturbations of Simulated Human Walking Depends on the Muscle Function
Source: Front Comput Neurosci. 2018 Oct 9;12:80. doi: 10.3389/fncom.2018.00080 (PMC6190627; doi:10.3389/fncom.2018.00080)
Supplement: Supplementary file 1 [file Data_Sheet_1.PDF]

Electronic supplementary material:
The benefit of combining neuronal feedback and feed-forward control for robustness in step down perturbations of simulated human walking depends on the muscle function

Daniel F.B. Haeufle[*], Birgit Schmortte[†], Hartmut Geyer[‡], Roy Müller[§], Syn Schmitt[¶]

August 2, 2018

In this electronic supplementary material, we describe in more detail the findings for the combination of feed-forward and feedback signals on the level of individual muscles.

1 Walking simulations

Table 1 shows the results of the walking simulations with different levels of feed-forward contribution.

In the following we will describe the behaviour in more detail.

1.1 m. vastus

Applying the combination of control strategies solely to m. vastus allows the compensation of higher ground level perturbations. More precisely, with pure feedback control, which correspond to the original model (Geyer and Herr, 2010), the maximum perturbation height was found to be $h = -0.03\,\text{m}$. By employing a feed-forward control with a percentage of 30% or 40%, the model has the ability to overcome larger perturbation heights of $h = -0.04\,\text{m}$ and $h = -0.05\,\text{m}$, respectively (see Tab. 1c and Anim. 1, electronic supplementary material). For all successful cases, the periodic walking pattern is

[*]Multi-Level Modeling in Motor Control and Rehabilitation Robotics, Hertie Institute for Clinical Brain Research and Center for Integrative Neuroscience, Eberhard-Karls Universität Tübingen, Germany, daniel.haeufle@uni-tuebingen.de
[†]Biomechanics and Biorobotics, Stuttgart Research Center for Simulation Sciences (SC SimTech) University of Stuttgart, Germany
[‡]Robotics Institute, Carnegie Mellon University, Pittsburgh, PA, USA
[§]Institute of Sport Science, Friedrich Schiller University of Jena and Department of Neurology and Department of Orthopedic Surgery, Klinikum Bayreuth GmbH, Germany
[¶]Biomechanics and Biorobotics, Stuttgart Research Center for Simulation Sciences (SC SimTech) and Department of Sports and Movement Science, University of Stuttgart, Germany

restored within two to three steps after the perturbation. However, a feed-forward contribution of more than 50% always results in unsuccessful walking, even for unperturbed walking (Anim. 2).

Figure 1 compares the m. vastus neural stimulation for different feed-forward contributions during the first stepping cycle after a perturbation of $h = -0.04\,\mathrm{m}$ (colored lines) to the pure feedback pattern of level walking (black solid line). One of the significant differences in handling a lowering ground is the fact that the combined control technique leads to earlier stimulation and strain of the muscle before the heel is contacting the ground (between ⇃1* and ⇃1, marked with circle in Fig. 1).

This is also visible in the comparison of Figs. 2a vs. b, where the reaction to increasing perturbations is shown. The reason for the earlier onset of the stimulation is, that the feed-forward pattern does not consider the increased duration of the flight phase, as it is triggered on the last unperturbed take-off. This earlier stimulation onset seems to be beneficial for perturbed walking. Without it (dashed blue line in Fig. 1), the peak muscle stimulation during the ground contact (between heel contact ⇃ and toe-off ↾3) is significantly higher than the unperturbed reference line (i.e., 0.49 compared to 0.35 in the unperturbed case). During the ankle push-off phase (between heel-off ↾2 and toe-off ↾3), the muscle stimulations of the successful simulations always lie above the reference line of the unperturbed walk.

The consequence of the perturbation is visible in the hip and knee joint angles and the respective joint angle velocities (Fig. 2c, d and Fig. 3). The effect of the longer flight phase, in comparison to the reference trajectory (black solid), is a delay in the flexion of the joint angles shortly after heel ground contact. In addition, the increased kinetic energy due to the lower ground contact becomes visible in the increased joint velocity shortly after touch down. This additional kinetic energy causes a lower (more flexed) knee angle during the early stance phase until heel take-off (↾2), which is only partially counteracted by the increased m. vastus stimulation (Fig. 2a). In the successful cases, this increased stimulation results in only small knee and hip angle deviations in the push-off phase (between ↾2 and ↾3).

1.2 Muscles at the hip joint

As the m. vastus is a knee extensor muscle, it can only indirectly influence the hip joint. The perturbation leads to an increase in hip and knee joint velocity shortly after heel contact (Fig. 3b and d, after ⇃1) indicating that the additional energy from the perturbation also affects the hip joint movement. This suggests, that also the muscles that directly influence the hip joint could contribute to the robustness. This was tested by adding feed-forward to the control of the hip flexor muscle group (HFL) or the m. gluteus (GLU) individually (see Tab. 1a and Tab. 1b). We find that no combination of the strategies increases the robustness in comparison to the pure feedback model, i.e., the maximum perturbation height remains $h = -0.03\,\mathrm{m}$. This means, that neither m. gluteus nor hip flexor muscle group alone are capable of dissipating the additional kinetic energy and recover from the perturbation by adding the feed-forward control strategy.

h [m]	Feed-forward ω [%]						
GLU	0	10	20	30	40	50	60
0.00	✓	✓	✓	✓	✓	✓	✓
-0.01	✓	✓	✓	✓	✓	✓	✓
-0.02	✓	✓	✓	✓	✓	×	×
-0.03	✓	✓	✓	✓	✓	✓	✓
-0.04	×	×	×	×	×	×	×

(a) GLU

h [m]	Feed-forward ω [%]				
HFL	0	10	20	30	40
0.00	✓	✓	✓	×	×
-0.01	✓	✓	✓	×	×
-0.02	✓	✓	✓	×	×
-0.03	✓	✓	✓	✓	×
-0.04	×	×	×	×	×

(b) HFL

h [m]	Feed-forward ω [%]						
VAS	0	10	20	30	40	50	60
0.00	✓	✓	✓	✓	✓	✓	×
-0.01	✓	✓	✓	✓	✓	✓	×
-0.02	✓	✓	✓	✓	✓	✓	×
-0.03	✓	✓	✓	✓	✓	✓	×
-0.04	×	×	×	✓	✓	×	×
-0.05	×	×	×	×	✓	×	×
-0.06	×	×	×	×	×	×	×

(c) VAS

h [m]	Feed-forward ω [%]				
SOL	0	10	20	30	40
0.00	✓	✓	✓	✓	×
-0.01	✓	✓	✓	✓	×
-0.02	✓	✓	✓	✓	×
-0.03	✓	✓	✓	✓	×
-0.04	×	✓	✓	✓	×
-0.05	×	✓	✓	×	×
-0.06	×	×	×	×	×

(d) SOL

perturb. height h [m]	Feed-forward ω [%]										
GAS	0	10	20	30	40	50	60	70	80	90	100
0.00	✓	✓	✓	✓	✓	✓	✓	✓	✓	✓	✓
-0.01	✓	✓	✓	✓	✓	✓	✓	✓	✓	✓	✓
-0.02	✓	✓	✓	✓	✓	✓	✓	✓	✓	✓	✓
-0.03	✓	✓	✓	✓	×	✓	✓	×	✓	✓	✓
-0.04	×	×	×	×	×	✓	✓	✓	✓	✓	✓
-0.05	×	×	×	×	×	×	✓	✓	✓	✓	×
-0.06	×	×	×	×	×	×	✓	×	✓	✓	×
-0.07	×	×	×	×	×	×	✓	×	✓	✓	×
-0.08	×	×	×	×	×	×	×	×	×	×	×

(e) GAS

Table 1: The tables a-e show successful (✓) and unsuccessful (×) walking simulations for variations in perturbation height and feed-forward contribution. GLU: m.gluteus, HFL: hip flexor muscle group, VAS: m.vastus, SOL: m.soleus, GAS: m.gastrocnemius.

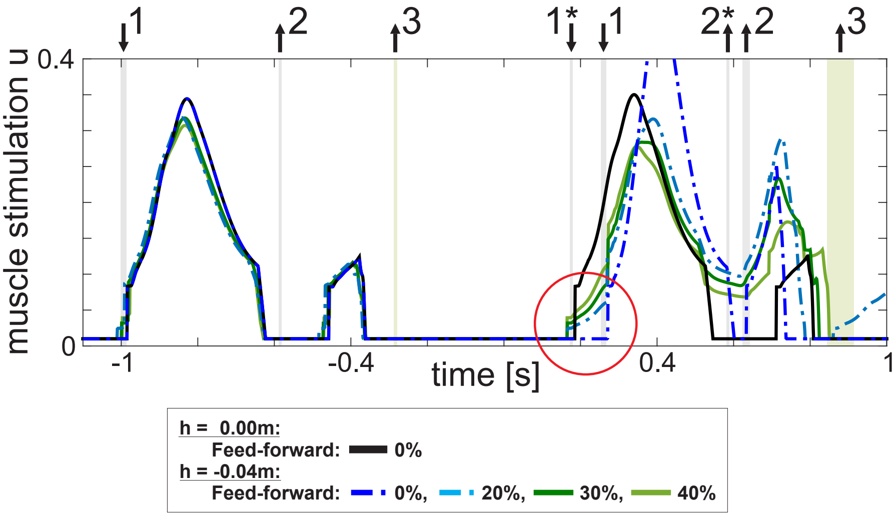

Figure 1: Stimulation u_{VAS} of the m. vastus (VAS) of the primarily perturbed leg for different combinations of feed-forward and feedback in the case of a $h = -0.04\,\text{m}$ perturbation in ground level. Solid lines represent successful cases (✓) and broken lines represent unsuccessful cases (×), where the model cannot continue to walk after the perturbation (Tab. 1c). The reference case (black solid) is the case of level walking ($h = 0\,\text{m}$) with pure feedback control (0%). Heel contact is indicated by ↓1, heel-off by ↑2, and toe-off by ↑3. Heel-contact and heel-off marked with * represent the reference case (0%, level walking). The time is synchronised to the toe-off of the contra-lateral leg ($t = 0$), which also serves as the trigger time for the feed-forward stimulation. The early ground contact phase after the perturbation is highlighted by the circle. By lowering the ground, the perturbation results in an extension of the swing phase of about 0.1 s as indicated by the shift between ↓1* and ↓1. Without feed-forward, the stimulation can only increase after the ground contact. However, with 40% feed-forward contribution, the stimulation onset is earlier and the peak stimulation around $t = 0.4\,\text{s}$ is lower.

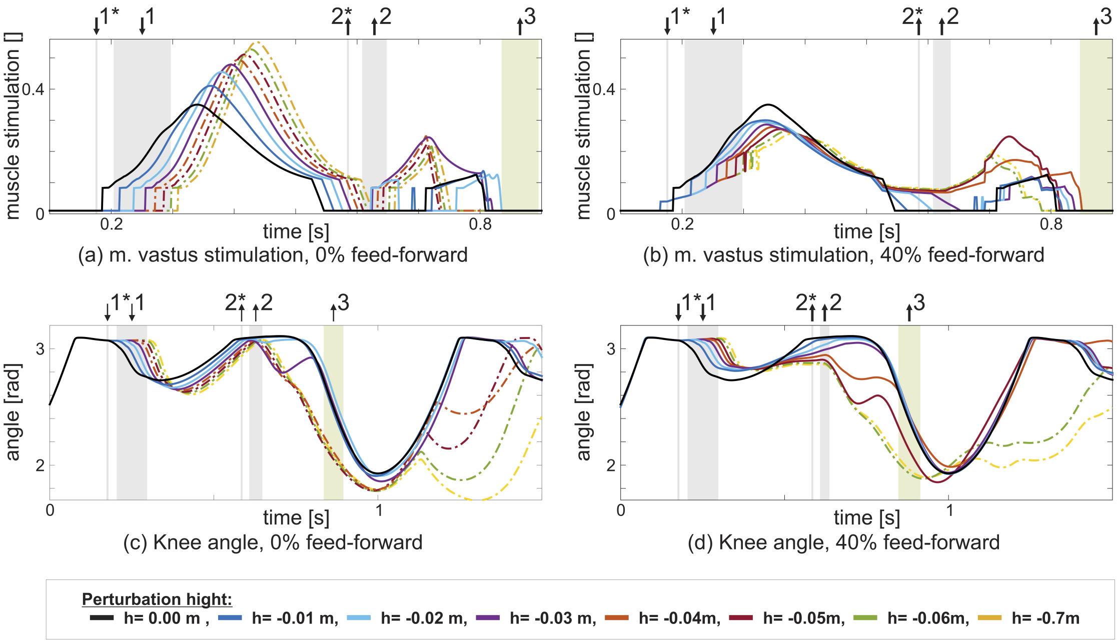

Figure 2: This figure shows the change in stimulation u_{VAS} and knee joint angle for increasing perturbation height h with the same feed-forward / feedback combination: 0% in the left column, and 40% in the right column. Step size increases from $h = 0\,\mathrm{m}$ (black) to $h = -0.07\,\mathrm{m}$. Heel contact is indicated by ↓1, heel-off by ↑2, and toe-off by ↑3. Heel-contact and heel-off marked with * represent the reference case (0%, level walking). It becomes visible, how an increased perturbation first delays and in consequence increases m. vastus stimulation, if no feed-forward contribution is present. The delayed stimulation causes the knee to further flex in the stance phase. In consequence, the model stumbles. For the combined control (40%), the delay is partially compensated by the triggered feed-forward stimulation pattern. Hence, the knee is less flexed and stumbling is prevented for larger perturbations.

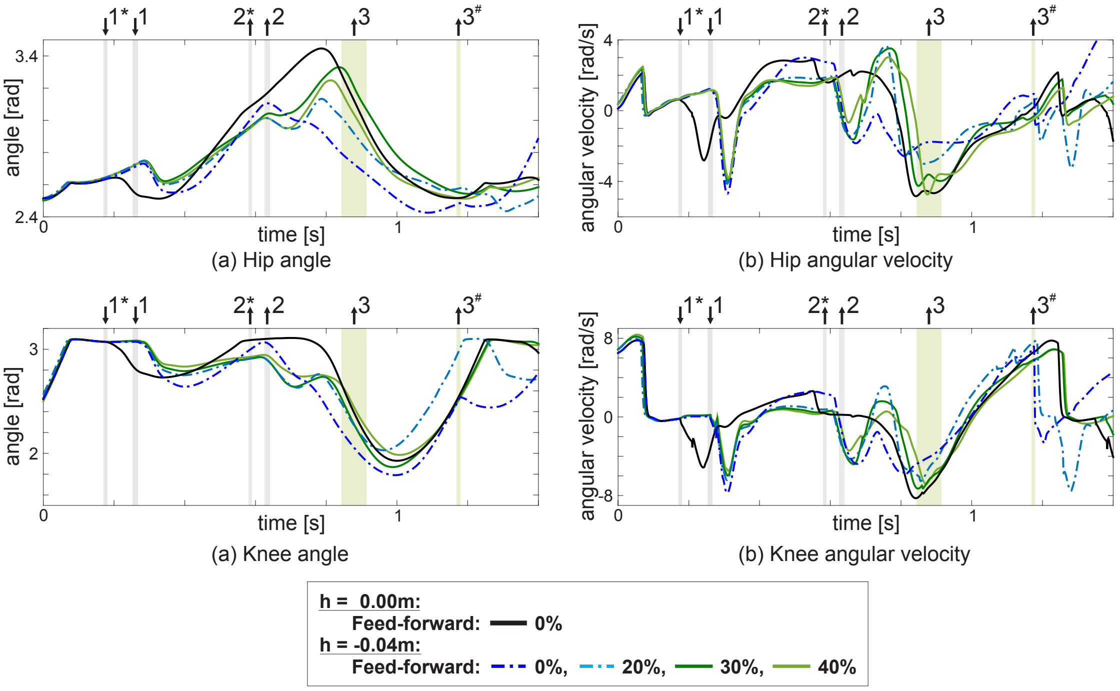

Figure 3: Joint angles and angular velocities of the primarily perturbed leg for different percentage combinations of feed-forward and feedback of m. vastus in the case of a $h = -0.04\,\mathrm{m}$ perturbation in ground level. The hip angle is defined as the angle between trunk and the thigh. The knee angle represents the angle between thigh and the shank. In upright position the hip and the knee angle equal π. Color scheme and control weightings are consistent with Fig. 1. Heel contact is indicated by $\downarrow$1, heel-off by $\uparrow$2, and toe-off by $\uparrow$3. Heel-contact and heel-off marked with * represent the reference case with 0% for level walking. The event at $\uparrow 3^{\#}$ is an unexpected toe-off in the case of 20%, indicating a stumbling step.

1.3 M. soleus

Looking at the ankle joint, a low feed-forward contribution of 10%-20% in the ankle extensor muscle m. soleus generates an earlier heel-off after the perturbation. This ankle extension lifts the center of mass and generates a higher ground clearance when the swing leg passes the ground. With this strategy, perturbations of up to $h = -0.05\,\mathrm{m}$ can be compensated (Anim. 3).

1.4 M. gastrocnemius

M. gastrocnemius is a bi-articular muscle flexing the knee and extending the ankle. We find that a medium feed-forward contribution of up to 40% to the stimulation of this muscle does not improve the maximum perturbation height (Tab. 1e). In these cases, the stance leg cannot generate enough ground clearance for the swing leg in the step after the perturbation causing a very short step length and, in consequence, stumbling. Higher feed-forward contributions of more than 60%, however, allow to compensate perturbations of up to $h = -0.07\,\mathrm{m}$. This is more than twice the normal maximum perturbation. In these cases, however, the walking pattern changes in several aspects. M. gastrocnemius stimulation onset is earlier after the perturbation resulting in a more extended leg during stance phase and an earlier heel lift-off. On the positive side, this allows the swing leg to pass through again. On the down-side, the push-off is less powerful and the peak knee joint velocity decreases. Therefore, with increasing feed-forward contribution in m. gastrocnemius, the entire walking pattern is perturbed and changes its rhythm (Anim. 4). This is visible, e.g., in a reduced walking speed.

1.5 Combining muscles

As stated above, the perturbation increases both hip and knee joint velocities. Therefore, we applied a combination of the most robust case for the m. vastus group (40% feed-forward) with the hip muscles. We find that adding more than 10% feed-forward control to the hip flexor muscle group actually destabilizes the movement (Tab. **??**). In contrast, a combination of 40% feed-forward in the m. vastus and 20% or 30% feed-forward in the m. gluteus actually increases the robustness – perturbations of up to $h = -0.06\,\mathrm{m}$ can be compensated (Tab. **??**, (Anim. 5)). Thus, in comparison to the pure feedback case, such a combination increases the possible perturbation height to 200%.

To investigate further improvements by muscle combinations, we systematically combined the muscles pairwise with all possible feed-forward contribution levels $\omega_{\mathrm{mus1}}, \omega_{\mathrm{mus2}} \in \{10\%, 20\%, ..., 90\%\}$. We found, that a combination of m. vastus (50%) and m. soleus (10%) generates robust walking for up to $h = -0.07\,\mathrm{m}$ with a natural walking pattern (Anim. 6). With m. soleus (20%) and the hip flexor group (40%), perturbations of up to $h = -0.09\,\mathrm{m}$ – three times the pure feed-forward robustness – can be compensated (Anim. 7). Here, the recovery after the perturbation takes several steps and the original walking pattern is not fully recovered within 10 seconds. But the walking pattern still looks natural.

Several combinations with high feed- forward contributions of m. gastrocnemius further improved the perturbation height. The most robust case was found for m. gastrocnemius (80%) m. gluteus (10%), which allowed to compensate for perturbations of up to $h = -0.12$ m. This corresponds to 400% of the pure feedback case. However, the high feed-forward contribution of the m. gastrocnemius in these cases also resulted in a slower and unnatural walking pattern, as described above (Anim. 8).

2 Hopping model

In previous simulation studies on simple one-dimensional hopping, it was shown that a combination of feed-forward and feedback allows to increase hopping stability (Haeufle et al., 2012). Increased stability in this one-dimensional scenario means that perturbations can be reduced in fewer hopping cycles as compared to either pure feed-forward or pure feedback control. This is visualized in the return-map of the hopping patterns (Fig. 4A). Here, a horizontal slope of the return map at the fixed point ($S = 0$) means that 100% of the perturbation is compensated within one hopping cycle. A horizontal slope of the return map can be achieved by combining feedback with feed-forward control.

The new simulations for the hopping model with knee joint and muscle-tendon characteristics predict a similar behavior (Fig. 4B). Both, pure feed-forward and pure feedback control generate stable hopping behavior with $S = -0.1$ and $S = 0.6$ respectively, but both do not achieve an optimal compensation ($S = 0$). However, the stability can be increased with a combination of the strategies. All combinations of feedback and feed-forward control result in stable hopping. A 50% contribution of feed-forward control actually allows to compensate small perturbations within one cycle ($S = 0$). This confirms the previous findings (Haeufle et al., 2012) and extends the conclusion to a biomechanically more realistic hopping model: A combination of feedback and feed-forward control of the antigravity muscles allows to increase stability with respect to external perturbations in rebounding behaviours.

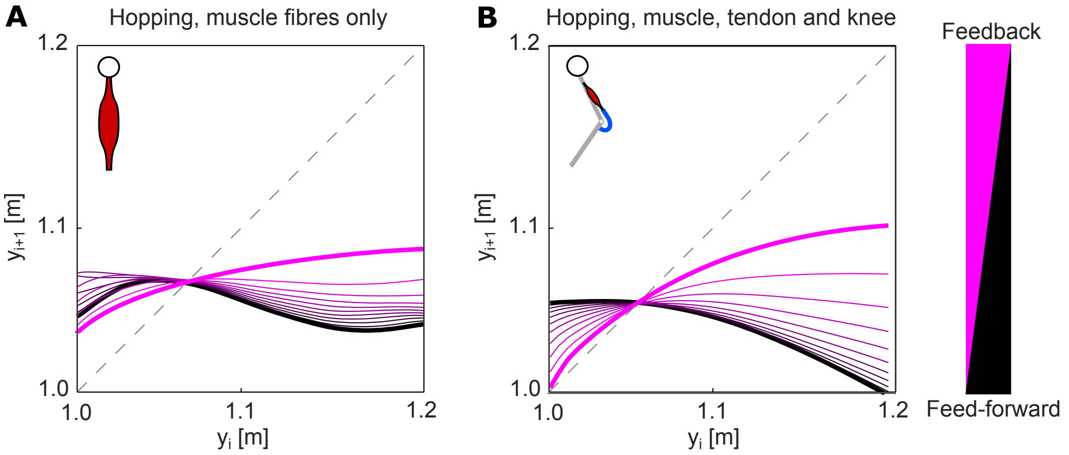

Figure 4: Return maps for the hopping models show the model reaction to perturbations: $y_{i+1}(y_i)$ is the relation between release height y_i and subsequent apex height y_{i+1}. Release height varied between $1.0\,\mathrm{m} < y_i < 1.2\,\mathrm{m}$. Intersections of return maps with the diagonals (dashed lines) indicate fixed points y_{fix} (periodic solutions with $y_{i+1} = y_i$). Here, all fixed points are stable, as the slope $S = dy_{1+1}/dy_i$ of the return map at the fixed point is always $|S| < 1$. Shown is a comparison of return maps with force-feedback (thick magenta line) and feed-forward (thick black line). The thin lines represent the return maps for the combination of both activation types under different weighting w as indicated by the color bar at the right: the brighter the lines are, the stronger is the contribution of the feedback (higher values for w). **A**: Return map of a hopping model driven by one muscle, but without any tendon elasticity or leg geometry. These results and the according model are described in detail elsewhere (Haeufle et al., 2010, 2012). **B:** Return map of a hopping model driven by one muscle, including tendon elasticity and knee joint. These new results confirm, that the combination of feedback and feed-forward improves stability, also in a more complex model. The model is described in detail elsewhere (Geyer et al., 2003).

References

Geyer, H. and Herr, H. A muscle-reflex model that encodes principles of legged mechanics produces human walking dynamics and muscle activities. *IEEE Transactions on Neural Systems and Rehabilitation Engineering*, 18(3):263–73, 2010. doi: 10.1109/TNSRE.2010.2047592.

Geyer, H., Seyfarth, A., and Blickhan, R. Positive force feedback in bouncing gaits? *Proceedings of the Royal Society of London. Series B*, 270(1529):2173–83, 2003. doi: 10.1098/rspb.2003.2454.

Haeufle, D. F. B., Grimmer, S., and Seyfarth, A. The role of intrinsic muscle properties for stable hopping - stability is achieved by the force-velocity relation. *Bioinspiration & Biomimetics*, 5(1):016004, 2010. doi: 10.1088/1748-3182/5/1/016004.

Haeufle, D. F. B., Grimmer, S., Kalveram, K.-T., and Seyfarth, A. Integration of intrinsic muscle properties, feed-forward and feedback signals for generating and stabilizing hopping. *Journal of the Royal Society, Interface*, 9(72):1458–69, 2012. doi: 10.1098/rsif.2011.0694.
